# Longitudinal Associations Between Work‐Related Factors and the Need for Recovery of Long‐Term Care Workers

**DOI:** 10.1111/nhs.70286

**Published:** 2026-01-04

**Authors:** Lise J. J. Dams, Ceciel H. Heijkants, Cécile R. L. Boot

**Affiliations:** ^1^ Radboud University, Behavioural Science Institute Nijmegen the Netherlands; ^2^ Amsterdam Public Health Research Institute, Societal Participation and Health Amsterdam the Netherlands; ^3^ Amsterdam UMC, VU University, Department of Public and Occupational Health Amsterdam the Netherlands

**Keywords:** long‐term care, NFR, quantitative job demands, social support, sustainable employability

## Abstract

Due to population aging, the need for long‐term care is increasing, while the workforce is decreasing. This leads to high workloads for those remaining in the workforce. An imbalance between job demands and job resources can lead to health problems through a high need for recovery (NFR). The aim of this study was to examine the longitudinal associations of work‐related factors with NFR. We performed a one‐year prospective cohort study of long‐term care workers (*n* = 108). Mixed models were used to analyze the longitudinal associations between quantitative job demands and social support from colleagues with NFR. The longitudinal effect of quantitative job demands on NFR was 13.82 (95% CI 9.59; 18.05). No effect was found for social support on NFR (−0.15 (95% CI −0.38; 0.08)). Quantitative job demands are associated with an increase in NFR over time. As job demands in long‐term care are difficult to reduce, we suggest improving job resources to create the needed balance between job demands and job resources to prevent a high NFR and to improve the sustainable employability of long‐term care workers.

**Trial Registration:** Netherlands Trial Register, NL9627.

## Summary

- What this paper adds
  ○ Need for recovery is associated with an imbalance between job demands and resources among long‐term care workers.
  ○ Quantitative job demands are associated with an increased need for recovery among long‐term care workers.
  ○ Mitigating job demands by increasing resources is recommended to reduce the need for recovery among long‐term care workers.

## 1 Introduction

Increased workload due to staff shortages in long‐term care is not a recent phenomenon. Ensuring sustainable employability of care workers in long‐term care is crucial, given the expected increase in the number of individuals needing long‐term care (Jurij et al. 2023). Sustainable employability refers to the conditions enabling employees to contribute through their work, now and in the future, while maintaining their health and well‐being (Van der Klink et al. 2016).

By 2050, more than half of Western Europe's population will be older than 48 years old, primarily due to the “baby boom” generation and improved healthcare (Dockery et al. 2015; Jurij et al. 2023). Consequently, there will be a larger group of older adults requiring increasingly complex care. At the same time, in the Netherlands, staff turnover in long‐term care facilities was 13.6% in 2020 (Consultancy.nl 2021), and the sickness absenteeism rate was above 8% in 2022 (CBS 2023). There is an increasing unfulfilled need for long‐term care, partly caused by staff turnover and sickness absenteeism. In addition, the number of long‐term care workers available is decreasing, resulting in a higher workload for a shrinking pool of staff (Hussein 2018; Jurij et al. 2023; Pélissier et al. 2018; Strandell 2020).

Chronic fatigue and burnout are common outcomes of long‐term work‐related stressors, such as continuous high workload (Schaufeli and Salanova 2024). Experiencing high levels of mental and physical fatigue after work, also known as a high need for recovery (NFR), can be considered a short‐term precursor of chronic fatigue and other health issues. The Job Demands‐Resources (JD‐R) Model provides an explanation for how specific work‐related factors may be related to NFR (Demerouti et al. 2001). The JD‐R Model distinguishes between job demands and job resources. *Job demands* are aspects of a job requiring continuous physical or mental effort and are thus associated with physiological and psychological costs such as work‐related fatigue (De Jonge et al. 2024; Demerouti et al. 2001). Quantitative job demands, including workload and work intensity, are considered central job demands (Van Veldhoven 2024). Van Veldhoven (2024) defined these quantitative aspects of job demands as “those elements of the work environment that concern the amount and speed of work to be performed within a given timespan, and that require physical and/or psychological effort.” (p. 151). *Job resources* are physical, psychological, social, or organizational aspects of the work environment that can mitigate the negative effects of job demands. Social support in the workplace is considered a key job resource and is characterized as helpful interactions with supervisors and co‐workers (Daniels et al. 2014). The JD‐R model also emphasizes that resources not only have a direct positive effect but can become particularly valuable in situations of high job demands, as they may help employees cope more effectively with these demands. A lack of job resources makes it harder for employees to meet job demands, which may lead to long‐term health problems (Demerouti et al. 2001; Diehl et al. 2021; Geurts et al. 2014; Hakanen et al. 2019; Schaufeli and Salanova 2024).

Longitudinal research has provided evidence that NFR is an important precursor of health outcomes. For instance, Sluiter et al. (2003) reported that higher levels of NFR predicted the development of subjective health complaints over time. Gommans et al. (2016) found that physical work demands were positively associated with NFR and adverse employment outcomes in their total sample. However, this association was not significant within the health care sector. Similarly, a cross‐sectional study of Ketels et al. (2022) reported positive associations between physically demanding tasks and NFR, but did not examine long‐term care settings specifically. A cross‐sectional study by Sun et al. (2021) examined this relationship and found that an imbalance between job demands and resources, such as support, was associated with higher NFR (Sun et al. 2021). Lastly, Clausen et al. (2014) also emphasized the importance of social support at work, in order to maintain or improve well‐being among long‐term care workers. As far as NFR was related to work‐related factors, most findings have been based on cross‐sectional designs, were measured at a single point in time, or were not specifically focused on long‐term care workers. To the best of our knowledge, this is one of the few studies that examines longitudinal associations between work‐related factors and NFR among care workers in long‐term care.

Therefore, the aim of the present study was to gain insight into the associations of work‐related factors, that is, quantitative job demands and social support from colleagues, with NFR over time. This study examines how changes in work‐related factors over the course of 1 year are associated with changes in NFR among long‐term care workers.

## 2 Materials and Methods

### 2.1 Design

This prospective cohort study used data from a randomized controlled trial (RCT) (Heijkants et al. 2022). Data were collected between 2021 and 2022 using questionnaires.

### 2.2 Setting

The study population consisted of care workers from one mid‐ to large‐sized Dutch healthcare organization. Half of the teams received a team‐based intervention to enhance their sustainable employability (the Healthy Working Approach). The goal of this intervention was to identify problems at team level, implement solutions, and evaluate if the problems were resolved. A detailed description of the intervention can be found in Heijkants et al. (2022). This study was performed in line with the principles of the Declaration of Helsinki. Approval was granted by the Ethics Committee of the Radboud University (No. ECSW‐2021‐012).

### 2.3 Sample

Long‐term care workers include all staff employed within the healthcare organization in various roles. We included care and well‐being personnel (e.g., nursing assistants or activities coordinators), physicians and practitioners (e.g., speech therapists or psychologists), and support services personnel (e.g., HR, IT, or kitchen). Inclusion criteria required long‐term workers to be able to read and understand the Dutch language and be aged 18 years or older. Exclusion criteria specified that long‐term care workers who had been on sick leave for 1 month or more prior to completing the baseline questionnaire and whose employment contract was ending within 6 months after completing the baseline questionnaire were not eligible. Care teams were invited by the researchers (Heijkants et al. 2022) to participate via internal communication tools, such as intranet and personal email. These invites included a link to the baseline questionnaire, starting with an eligibility check and a digital consent.

To be included in the analysis long‐term care workers needed to have values on all core variables at one or more time points. One participant did not meet this criterion, so from *n* = 108 participants, *n* = 107 were included in the analysis.

### 2.4 Measures

#### 2.4.1 Dependent Variable: Need for Recovery (NFR)

NFR is the extent to which one needs to recover from work‐induced fatigue. NFR was measured by the questionnaire on Psychosocial Job Demands and Job Stress (Van Veldhoven and Meijman 1994). The variable contains 11 dichotomous items with “no” or “yes” answer options, assessing the extent to which an individual feels physically and mentally exhausted after work. One of the questions was, for example, “It generally takes me more than an hour before I am fully recovered after work.” The 11 scores on NFR were summed into a total score for each time point. This total score per time point was then converted to a scale from 0 to 100 per time point, with higher scores indicating higher NFR.

#### 2.4.2 Independent Variables: Job Demands and Social Support From Colleagues

*Quantitative job demands* refer to the amount and speed of work to be performed within a given timespan (Van Veldhoven 2024). Quantitative job demands were measured by 3 items on a 4‐point scale ranging from never to always (1–4) derived from the Dutch Survey of Working Conditions (Hooftman et al. 2021). A mean score was calculated for the 3 items, with a higher score implying higher quantitative job demands.

*Social support from colleagues* refers to the frequency of receiving assistance from colleagues, their willingness to listen to problems, and their acknowledgment of job performance. Social support from colleagues was measured with 3 items from the Dutch version of the Copenhagen Psychosocial Questionnaire (COPSOQ) (Burr et al. 2019). Items were measured on a 5‐point scale ranging from never to always (1–5), after which they were converted to a scale of 0–100 per item. Subsequently, a mean score was calculated for each time point; a higher score means more social support from colleagues.

#### 2.4.3 Potential Confounders

The following potential confounders were taken into account**:** Age (years), Informal caregiving (hours), Employment years (< 1 year; 1–10 years; > 10 years), Working hours per week (<20 h; 20–28 h; >28 h), Type of contract (Permanent; Temporary), Role in organization (Care and well‐being; Physicians and practitioners; Support services), Highest level of education (Primary/pre‐vocational secondary; Senior general secondary/University preparation education/vocational; Higher vocational/university), Self‐reported health (0–100) and Physical demands (1–3).

### 2.5 Analytic Strategy

Descriptive analyses were conducted to describe the sample. Prior to running the analyses, the following assumptions were checked: linearity, normality of residuals, homoscedasticity, and the normality of the intercepts and slopes. A linear mixed model analysis was used to examine the longitudinal associations between the work‐related factors and NFR. This multilevel approach takes the correlation within multiple levels into account: multiple time points (i), individual long‐term care workers (j), and care teams (k). Since each care worker has multiple time point measurements, it can be assumed that the measurements within each care worker correlate strongly; therefore, a random intercept at the individual level (care worker: j) was applied in the analysis. Since the Healthy Working Approach was implemented in half of the participating teams, although this is not the focus of the present study, we added group membership (intervention/control group) as a confounder to the final models. For each possible confounder a sequential adjustment method was applied: a confounding variable was considered relevant if its inclusion in the model changed the effect estimate of the independent variable by more than 10%. We performed a sensitivity analysis with complete cases (54 out of 107 participants filled out the questionnaires at all four time points) to test the robustness of the findings. In addition, we tested a possible moderation effect by adding an interaction term (quantitative job demand × social support) to the model. In case of a significant interaction, the interaction term will be included in the main model. Data were cleaned and explored in the software program IBM SPSS statistics 27 and analyzed in the software program Stata 17.0.

## 3 Results

### 3.1 Sample

The total sample consisted of 107 long‐term care workers, of which 54 responded to all follow‐up measurements (referred to as “complete cases”). Participants completed the questionnaire containing measures of work‐related factors and NFR at baseline (*n* = 105), and after 6 months (*n* = 86), 9 months (*n* = 68), and after 12 months (*n* = 67). Table 1 shows that 70% of the long‐term care workers were between 18 and 54 years of age. Additionally, over 90% of the care workers were women. Most of the long‐term care workers had obtained a senior general secondary‐, pre‐university‐ or vocational education (> 40%), or a higher vocational‐ or university education (> 40%) as the highest level of degree. In addition, most of the long‐term care workers worked 28 contractual hours or less per week (57%). Lastly, examining the core variables presented in Table 2 shows that the baseline mean score of NFR is 31.7, which increases to 43.4 after a 12‐month period.

**TABLE 1:** Study population described by sociodemographic factors.

	Total sample (*n* = 107)
Age	
< 55 years old, *n* (%)	75 (70.1)
Gender	
Female, *n* (%)	98 (91.6)
Highest level of education	
Primary/pre‐vocational secondary^a^, *n* (%)	19 (17.8)
Senior general secondary/university preparation education/vocational^b^, *n* (%)	44 (41.1)
Higher vocational/university^c^, *n* (%)	44 (41.1)
Role in organization	
Care and well‐being, *n* (%)	83 (77.6)
Physicians and practitioners, *n* (%)	11 (10.3)
Support services, *n* (%)	13 (12.1)
Type of contract	
Permanent, *n* (%)	90 (84.1)
Temporary, *n* (%)	17 (15.9)
Years employed	
< 1 year, *n* (%)	19 (17.9)
1–10 years, *n* (%)	35 (33.0)
> 10 years, *n* (%)	52 (49.1)
Working hours per week	
≤ 28 h, *n* (%)	61 (57.0)

^a^ In Dutch: basisonderwijs/MAVO.

^b^ In Dutch: HAVO/VWO/MBO.

^c^ In Dutch: HBO/WO.

**TABLE 2:** Study population described by the core variables.

Core variables of present study	T0 (*n* = 105)	T6 (*n* = 86)	T9 (*n* = 68)	T12 (*n* = 67)
Need for recovery, mean (SD)	31.7 (26.5)	36.9 (28.6)	37.3 (27.7)	43.4 (31.5)
Quantitative job demands, mean (SD)	2.2 (0.5)	2.2 (0.6)	2.3 (0.6)	2.3 (0.6)
Social support, mean (SD)	70.7 (13.0)	67.8 (13.8)	68.3 (13.3)	69.3 (15.2)

### 3.2 Linear Models

The results of the linear mixed model analyses are presented in Table 3. The overall longitudinal effect of quantitative job demands on NFR is 13.82 (95% CI 9.59; 18.05). This significant overall effect (*p* < 0.01) indicates that with every unit difference in quantitative job demands, there is a significant increase of 13.82 in NFR. Given the longitudinal analysis this effect has a double interpretation, as it reflects a within subject and between subjectseffect. When quantitative job demands increase one unit somewhere over time within one care worker, NFR increases with 13.82 (within subjects). When quantitative job demands of care worker A are one unit higher than the quantitative job demands of care worker B, NFR score is 13.82 higher for care worker A in comparison to care worker B (between subjects).

**TABLE 3:** Longitudinal effects of quantitative job demands and social support on need for recovery.

Need for recovery	Quantitative job demands			Social support from colleagues		
	Crude model (*n* = 107)^a^	Adjusted model (*n* = 107)^b^	Sensitivity analysis (*n* = 54)^b^	Crude model (*n* = 107)^a^	Adjusted model (*n* = 107)^b^	Sensitivity analysis (*n* = 54)^b^
Overall effect, *ϕ3* (95% CI)	13.61 (9.38; 17.83)**	13.82 (9.59; 18.05)**	13.00 (7.98; 18.03)**	−0.15 (−0.38; 0.08)	−0.15 (−0.38; 0.08)	−0.10 (−0.37; 0.18)

^a^ Not adjusted for *Group* (intervention/control).

^b^ Adjusted for *Group* (intervention/control), no other relevant confounders were found.

* *p* < 0.05.

** *p* < 0.01.

The overall longitudinal effect between social support from colleagues and NFR is −0.15 (95% CI −0.38; 0.08). This indicates that an increase in social support from colleagues was not significantly associated with a decrease in NFR. No significant interaction effect was found for social support × quantitative job demands.

### 3.3 Sensitivity Analysis

The robustness of the findings was tested with a sensitivity analysis with complete cases. The results showed that the longitudinal effect of quantitative job demands on NFR was maintained (β *=* 13.00, *p* < 0.01), irrespective of the relatively small sample size (*n* = 54) (Table 3).

## 4 Discussion

The results showed a positive significant overall longitudinal effect of quantitative job demands on NFR, indicating that an increase in quantitative job demands is significantly associated with increasing levels of NFR over time. Furthermore, social support showed no significant overall longitudinal relation with NFR. The finding that high quantitative job demands are associated with high NFR over time is in line with previous findings. For instance, Gommans et al. (2016) found in a longitudinal design that physical work demand was positively associated with NFR. Our findings further support the importance of NFR given its predictive value for later health complaints (Sluiter et al. 2003). Sun et al. (2021) described that high NFR significantly affected a physician's well‐being due to an imbalance between (high) job demands and (low) job resources. Furthermore, Aronsson et al. (2017) described in their systematic review how high job demands were associated with the development of burnout, which can be considered a long‐term outcome of persistent high NFR. Although findings align with previous research, the direction of the association remains unclear. For example, NFR may also be related to perceived workload, through fatigue‐related perception bias (Guthier et al. 2020).

The findings related to social support of the present study are not consistent with findings from previous studies. For instance, Aronsson et al. (2017) demonstrated a protective relationship between job support and burnout. Also, other scholars emphasized the importance of (social) support to reduce NFR (Sun et al. 2021) and turnover (Clausen et al. 2014) among healthcare workers and argued that positive encouragement and support would improve their workplace well‐being.

The present study could not demonstrate a longitudinal association between social support from colleagues and NFR. These findings can be explained in different ways. One explanation relates to the overall high mean score on social support from colleagues (69 on a 0–100 scale). Not many long‐term care workers experienced low social support from colleagues (< 14%). However, sufficient variation in high and low scores on social support is a precondition for finding a relationship between social support and NFR. Another possible explanation relates to the measurement of social support and its outcome variables. For instance, Clausen et al. (2014) did not measure social support with the Copenhagen Psychosocial Questionnaire (COPSOQ), but measured how much long‐term care workers indicated that *more support and recognition from my colleagues* would have been a reason for them to stay in their jobs. This qualitative approach highlights the possibility of a buffer effect, whereby perceived social support from colleagues may serve as a protective factor against leaving their jobs. While these qualitative evaluations provide valuable insights into the perceived importance of social support from colleagues, they cannot reflect the more nuanced dynamics needed to demonstrate protective effects on NFR.

### 4.1 Methodological Considerations

A strength of the present study is its insight into the sustainable employability of a vital occupational group that has been under increasing pressure for years, and about whom little was known regarding work‐related factors and NFR. The longitudinal design is a strength as previous studies relied on cross‐sectional insights into the relationship between work‐related factors and NFR. By performing mixed model analyses, we took the correlation within multiple levels of the dataset into account. In addition, mixed models, compared to Generalized Estimating Equations (GEE), handle continuous dependent data with missing values more effectively (Twisk 2013).

However, using intervention study data also had its limitations. Only the long‐term care workers who wanted to participate in the RCT were selected, which means selection bias cannot be ruled out. For example, long‐term care workers with high quantitative job demands and/or high need for recovery could have been less likely to participate, because they had limited time and/or energy available to participate in an intervention study. A second limitation of the present study is the time period during which the data was collected. Data was collected during the (aftermath of the) COVID‐19 pandemic; a time that differed from other time periods. Because of higher sickness absenteeism during this period, more stress could have occurred among the care workers. Higher stress levels could have possibly been caused by higher workload, due to higher sickness absence among colleagues, related to COVID‐19. In addition, care workers may have experienced anticipatory stress related to getting sick due to COVID‐19, whether for themselves, their colleagues, their residents or their family and friends. Reme et al. (2023) concluded that the increased sickness absenteeism among health workers during the pandemic was not due to having to treat COVID‐19 patients, but rather due to health workers becoming infected with SARS‐CoV‐2 and dropping out as a result, and/or to increased workload caused by strict infection control measures. COVID‐19 likely contributed to the observed increase in NFR from baseline to 12 months. Other circumstances (such as relocations of teams and the introduction of small‐scale living in the healthcare organization) were also mentioned in the process evaluation of the accompanying RCT from which the data for this article were used (van Kuppenveld et al. 2025). These contextual factors could have placed additional strain on long‐term care staff; however, as we did not measure these factors directly, this explanation remains speculative. The higher workload and possibly related elevated levels of NFR during this period could have led to a stronger association (overestimation of the effect) within this period compared to other time periods. A third limitation is that the results should be taken into careful consideration because of the low generalizability; a relatively small number of care workers participated (*n* = 107), with data obtained from only one long‐term care facility. However, the present study included a broad range of long‐term care workers of different ages and with various roles within the organization, resulting in a heterogeneous study population.

### 4.2 Recommendations for Research and Practice

This study offers several research recommendations. Firstly, it can be questioned whether NFR was the only precursor of long‐term health problems in long‐term care workers, or whether there would have been additional precursors, such as work‐related fatigue or short‐term sickness absence. It may therefore be interesting to investigate how work factors would relate to other potential precursors of long‐term health problems among long‐term care workers.

Secondly, in the present study, social support was measured from colleagues without distinguishing between different forms of social support. Moreover, there are other forms and sources of social support, such as emotional or instrumental support and social support from supervisors or management, that could have been more important. Future research should examine NFR in relation to different forms and sources of social support.

Since data was collected from one healthcare organization, collecting data from more healthcare organizations would increase the generalizability of the findings. A next step to obtain more insight into the causal relationship between work characteristics and NFR would be to analyze time lags, which would require a longer follow‐up duration.

## 5 Conclusion

The purpose of the present study was to contribute to knowledge on improving the sustainable employability of care workers in long‐term care, by gaining insight into the relationships between work‐related factors and NFR over time. In conclusion, high quantitative job demands were significantly associated with a higher NFR over time. Improving the balance between job demands and resources is important to improve long‐term care workers' sustainable employability. The present study is the first to provide insight into the longitudinal relations between a selection of work‐related factors and NFR. However, further research needs to be conducted to develop and implement suitable interventions focusing on job resources and coping with job demands to effectively improve sustainable employability in long‐term care.

### 5.1 Recommendations for Clinical Practice

Findings from the present study emphasize the importance of lowering quantitative job demands to reduce high NFR. However, as it is rather difficult to reduce quantitative job demands in long‐term care due to chronic staff shortages, it is important to complement the imbalance between job demands and job resources by increasing job resources, such as autonomy, team cohesion, and coaching and support from the management. These resources can create a buffer for dealing with high quantitative job demands, and consequently reduce the NFR.

## Author Contributions

All authors were involved in the conceptual framework and analyses. Lise Dams wrote the first draft. Ceciel Heijkants and Cécile Boot provided feedback on multiple drafts and approved the final draft before submission.

## Funding

The authors have nothing to report.

## Ethics Statement

This study was performed in line with the principles of the Declaration of Helsinki. Approval was granted by the Ethics Committee of the Radboud University Nijmegen, the Netherlands (No. ECSW‐2021‐012).

## Consent

Informed consent was obtained from all individual participants included in the study.

## Conflicts of Interest

The authors declare no conflicts of interest.

## Data Availability

The data that support the findings of this study are available from the corresponding author upon reasonable request.
